# Inactivation of agmatinase expressed in vegetative cells alters arginine catabolism and prevents diazotrophic growth in the heterocyst-forming cyanobacterium *Anabaena*

**DOI:** 10.1002/mbo3.207

**Published:** 2014-09-10

**Authors:** Mireia Burnat, Enrique Flores

**Affiliations:** 1 Instituto de Bioquímica Vegetal y Fotosíntesis, Consejo Superior de Investigaciones Científicas and Universidad de SevillaAmérico Vespucio 49, E-41092, Seville, Spain

**Keywords:** Agmatinase, *Anabaena*, arginine catabolism, nitrogenase

## Abstract

Arginine decarboxylase produces agmatine, and arginase and agmatinase are ureohydrolases that catalyze the production of ornithine and putrescine from arginine and agmatine, respectively, releasing urea. In the genome of the filamentous, heterocyst-forming cyanobacterium *Anabaena* sp. strain PCC 7120, ORF *alr2310* putatively encodes an ureohydrolase. Cells of *Anabaena* supplemented with [^14^C]arginine took up and catabolized this amino acid generating a set of labeled amino acids that included ornithine, proline, and glutamate. In an *alr2310* deletion mutant, an agmatine spot appeared and labeled glutamate increased with respect to the wild type, suggesting that Alr2310 is an agmatinase rather than an arginase. As determined in cell-free extracts, agmatinase activity could be detected in the wild type but not in the mutant. Thus, *alr2310* is the *Anabaena speB* gene encoding agmatinase. The Δ*alr2310* mutant accumulated large amounts of cyanophycin granule polypeptide, lacked nitrogenase activity, and did not grow diazotrophically. Growth tests in solid media showed that agmatine is inhibitory for *Anabaena*, especially under diazotrophic conditions, suggesting that growth of the mutant is inhibited by non-metabolized agmatine. Measurements of incorporation of radioactivity from [^14^C]leucine into macromolecules showed, however, a limited inhibition of protein synthesis in the Δ*alr2310* mutant. Analysis of an *Anabaena* strain producing an Alr2310-GFP (green fluorescent protein) fusion showed expression in vegetative cells but much less in heterocysts, implying compartmentalization of the arginine decarboxylation pathway in the diazotrophic filaments of this heterocyst-forming cyanobacterium.

## Introduction

Cyanobacteria are oxygenic photosynthetic prokaryotes that constitute a phylogenetically coherent group of organisms (Giovannoni et al. 1988). However, they show very diverse morphologies, presenting both unicellular and multicellular forms (Rippka et al. 1979). Cyanobacteria such as those of the genera *Anabaena* and *Nostoc* grow as filaments of cells (trichomes) that, when incubated in the absence of a source of combined nitrogen, present two cell types: vegetative cells that fix CO_2_ performing oxygenic photosynthesis and heterocysts that carry out N_2_ fixation (Flores and Herrero 2010). Heterocysts differentiate from vegetative cells in a process that involves execution of a specific program of gene expression and intercellular transfer of regulators (Herrero et al. 2013). In the N_2_-fixing filament, heterocysts are spaced along the filament and provide the vegetative cells with fixed nitrogen; for this, heterocysts need to receive in turn photosynthate from the vegetative cells (Wolk et al. 1994). An intercellular exchange of glutamine for glutamate (Thomas et al. 1977; Martín-Figueroa et al. 2000) and transfer of *β*-aspartyl-arginine (Burnat et al. 2014) can move fixed nitrogen from heterocysts to vegetative cells. Conversely, alanine (Jüttner 1983; Pernil et al. 2010) and, mainly, sucrose (Jüttner 1983; Schilling and Ehrnsperger 1985; Curatti et al. 2002; López-Igual et al. 2010; Vargas et al. 2011) can transfer reduced carbon from vegetative cells to heterocysts.

Heterocysts conspicuously accumulate cyanophycin (multi-l-arginyl-poly [l-aspartic acid]), a nitrogen-rich reserve polymer (Lang et al. 1972; Sherman et al. 2000), which is seen as refractile granules that are located at the heterocyst poles (close to and inside the heterocyst “necks”). Although production of cyanophycin is not required for diazotrophic growth (Ziegler et al. 2001; Picossi et al. 2004), its conspicuous presence in the heterocysts suggests a possible role in diazotrophy, likely as dynamic nitrogen storage (Carr 1983; Haselkorn 2007). Cyanophycin is synthesized by cyanophycin synthetase and degraded by cyanophycinase, which releases *β*-aspartyl-arginine (Richter et al. 1999). In the diazotrophic filaments, both cyanophycin synthetase and cyanophycinase are present at high levels in the heterocysts (Gupta and Carr 1981a; Picossi et al. 2004). The *β*-aspartyl-arginine dipeptide is hydrolyzed to aspartate and arginine by an isoaspartyl dipeptidase (Hejazi et al. 2002), which in the model heterocyst-forming cyanobacterium *Anabaena* sp. strain PCC 7120 (hereafter *Anabaena*) has been found to be expressed preferentially in vegetative cells (Burnat et al. 2014). This implies that the dipeptide released by cyanophycinase in the heterocyst is transferred to vegetative cells serving as a nitrogen vehicle in the diazotrophic filament. The isoaspartyl dipeptidase products, arginine and aspartate, must be further catabolized to make their nitrogen atoms available for cellular metabolism.

**Figure 1:**
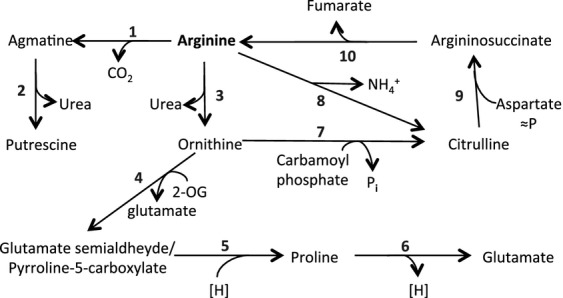
Schematic representation of the arginine decarboxylase, arginase and arginine deiminase pathways. Enzymes and the possible corresponding ORFs in the genome of *Anabaena* sp. strain PCC 7120 (Kaneko et al. 2001) are as follows: 1, arginine decarboxylase (*all3401*); 2, agmatinase (*alr2310*); 3, arginase; 4, ornithine transaminase (*alr1080*); 5, Δ^1^pyrroline-5-carboxylate reductase (*alr0488*); 6, proline oxidase (*alr0540*); 7, ornithine carbamoyltransferase (*alr4907*); 8, arginine deiminase; 9, argininosuccinate synthetase (*alr4798*); 10, argininosuccinate lyase (*alr3887*). Note that no gene is annotated as encoding arginase or arginine deiminase. 2-OG, 2-oxoglutarate; [H], reducing power; ≈P, energy-requiring reaction (energy provided by the hydrolysis of ATP).

Arginine serves as a source of nitrogen, carbon, and energy in different bacteria, and five possible catabolic pathways have been detected in cyanobacteria by bioinformatic analysis (Schriek et al. 2007). Of these, three (shown schematically in Fig.1) have been detected by physiological studies: the arginase pathway, the arginine deiminase pathway, and the arginine decarboxylase pathway (Quintero et al. 2000; Schriek et al. 2007; Weathers et al. 2010). In the arginase pathway, arginine is converted to ornithine releasing urea, and ornithine is further metabolized to glutamate (Cunin et al. 1986). Arginine deiminase produces citrulline and ammonium, with citrulline being further catabolized producing ornithine and carbamoyl phosphate (Cunin et al. 1986). In the arginine decarboxylase pathway, arginine is decarboxylated to agmatine (1-[4-aminobutyl]guanidine), which is then metabolized to putrescine (1,4-diaminobutane) releasing urea and further to 4-aminobutyrate and succinate (Cunin et al. 1986). In *Aphanocapsa* 6308 (currently known as *Synechocystis* sp. strain PCC 6308), it was reported that, besides the arginase pathway, which would provide only nitrogen for the cells, the arginine deiminase (also known as arginine dihydrolase) pathway provides carbon, nitrogen, and energy (Weathers et al. 1978). In *Synechocystis* sp. strain PCC 6803, based on in vivo studies with ^14^C-labeled substrates and mutational analysis, a model for arginine catabolism was proposed involving an arginase-like pathway combined with a urea cycle that would at the same time degrade aspartate (Quintero et al. 2000). The activity of arginine decarboxylase was also detected, but it was apparently less important than the arginase-like route under the experimental conditions investigated (Quintero et al. 2000). On the other hand, in a large-scale proteomic study of *Synechocystis* sp. strain PCC 6803, the enzymes of the arginine decarboxylation pathway (arginine decarboxylase and agmatinase) were observed to be up-regulated under certain environmental perturbations (Wegener et al. 2010). The arginine decarboxylase pathway, including conversion of putrescine to succinate, might serve as a source of carbon and nitrogen, as it has been described in *Escherichia coli* and *Pseudomonas* sp. (Kurihara et al. 2005; Chou et al. 2008). However, bioinformatic analysis of the genomes of 24 cyanobacteria (including *Synechocystis* sp. strain PCC 6803) failed to identify genes encoding putrescine oxidase or putrescine transaminase (needed to produce 4-aminobutyraldehyde in the arginine decarboxylase pathway that ends in succinate), suggesting that the arginine decarboxylase pathway in cyanobacteria could be mainly involved in the synthesis of polyamines and the production of ammonium from arginine (Schriek et al. 2007). Additionally, an amino acid oxidase with specificity for basic amino acids (especially arginine) that can release ammonium for growth is present in some cyanobacteria (Flores et al. 1982; Gau et al. 2007). On the other hand, arginine:glycine amidinotransferases involved in secondary metabolism have been described in some cyanobacteria (Muenchhoff et al. 2010; Barón-Sola et al. 2013).

In the *Anabaena* genome, genes encoding arginine decarboxylase and enzymes corresponding to the second and subsequent steps of the arginase pathway can be identified (Kaneko et al. 2001; see Fig.1). On the other hand, although the presence of an arginase-encoding gene is not evident, open reading frame (ORF) *alr2310* is annotated as “similar to agmatinase” (Kaneko et al. 2001). Arginases and agmatinases belong to the ureohydrolase protein family, which has led to wrong genomic annotations in the predicted functions of some ureohydrolase family proteins from different organisms, as has been the case for *Synechocystis* sp. strain PCC 6803 (Sekowska et al. 2000; see also Quintero et al. 2000). Therefore, it may be asked whether the product of ORF *alr2310* is an agmatinase or an arginase. In this work, we created and characterized *alr2310* mutants of *Anabaena* including deletion and reporter-expressing strains. We found that *alr2310* encodes an agmatinase that is mainly present in vegetative cells and is required for diazotrophic growth.

## Materials and Methods

### Strains and growth conditions

*Anabaena* sp. (also known as *Nostoc* sp.) strain PCC 7120 was grown axenically in BG11 medium (containing NaNO_3_), BG11_0_ medium (free of combined nitrogen) or BG11_0_ NH_4_^+^ medium (BG11_0_ containing 4 mmol/L NH_4_Cl and 8 mmol/L 2-[Tris(hydroxymethyl)-methylamino]-ethanesulfonic acid (TES)-NaOH buffer, pH 7.5). In every case, ferric citrate replaced the ferric ammonium citrate used in the original receipt (Rippka et al. 1979). For plates, medium was solidified with 1% separately autoclaved Difco agar. Cultures were grown at 30°C in the light (25 *μ*E m^−2^ sec^−1^), with shaking (80–90 rpm) for liquid cultures. Alternatively, cultures (referred to as bubbled cultures) were supplemented with 10 mmol/L of NaHCO_3_ and bubbled with a mixture of CO_2_ and air (1% v/v) in the light (50–75 *μ*E m^−2^ sec^−1^). For mutants described below, antibiotics were used at the following concentrations: erythromycin (Em), 5 *μ*g mL^−1^ for liquid cultures and 10* μ*g mL^−1^ for solid media; streptomycin sulfate (Sm) and spectinomycin dihydrochloride pentahydrate (Sp), 5 *μ*g mL^−1^ each for both liquid and solid media; and neomycin sulfate (Nm), 40 *μ*g mL^−1^ for solid media. DNA was isolated from *Anabaena* sp. by the method of Cai and Wolk (1990). *Anabaena* sp. strain FQ163, the *hepP* mutant (López-Igual et al. 2012), was grown in BG11 medium, supplemented when appropriate with antibiotics. All *Anabaena* strains used in this work are listed in Table S1.

*Escherichia coli* DH5*α* was used for plasmid constructions. It and strains HB101 and ED8654, used for conjugations with *Anabaena* sp., were grown in Luria–Bertani medium supplemented when appropriate with antibiotics at standard concentrations.

### Plasmid construction and genetic procedures

Open reading frame *alr2310* of the *Anabaena* chromosome (Kaneko et al. 2001) was inactivated by deleting an internal fragment of 927 bp. DNA fragments upstream (667 bp) and downstream (501 bp) from the central region of the gene were amplified by standard PCR using as template DNA from *Anabaena* and primers alr2310-3/alr2310-6 and alr2310-4/alr2310-5 (all oligodeoxynucleotide primers are listed in Table S2). The external primers alr2310-3 and alr2310-4 included SacI-sites in their 5′ ends and primers alr2310-5 and alr2310-6 included EcoRV-sites in their 5′ ends. The upstream and downstream DNA fragments from this gene were cloned in pMBL-T (Dominion MBL, Córdoba, Spain), sequenced, and transferred as a SacI-ended fragment to SacI-digested pCSBN1 (which is a plasmid derived from pCSV3 and pRL278 containing a Nm^R^ gene cassette and the *sacB* gene for positive selection), producing pCSBN5 (all plasmids are listed in Table S1).

Conjugation of *Anabaena* with *E. coli* HB101 carrying the cargo plasmid (pCSBN5) with helper and methylation plasmid pRL623 was effected by the conjugative plasmid pRL443, carried in *E. coli* ED8654, and performed as described (Elhai et al. 1997) with selection for resistance to Nm. Filaments of eight Nm^R^ clones were spread on BG11_0_ NH_4_^+^ medium supplemented with 5% sucrose (Cai and Wolk 1990), and individual Suc^R^ colonies were checked by PCR looking for clones that had substituted the wild-type locus by a deleted locus. The genetic structure of selected clones was studied by PCR with DNA from those clones and primers alr2310-3/alr2310-7 and alr2310-3/alr2310-8. A clone homozygous for the mutant chromosomes was named strain CSMI11 (Δ*alr2310*).

The plasmid carrying fusion gene *alr2310*-*gfp* was prepared as follows. A 681-bp fragment from the 3′-terminal part of *alr2310* was amplified by PCR using primers alr2310-11 and alr2310-13 (which lacks the stop codon of the gene and contains a sequence encoding a 4-Gly linker and a NheI site in its 5′ end) and *Anabaena* DNA as template, and the resulting fragment was cloned in vector pMBL-T producing plasmid pCSMI42. This fragment was corroborated by sequencing and transferred as a SacI/NheI-ended fragment to SacI/NheI-digested pCSAL33 (which contains, cloned in vector pMBL-T, the *gfp-mut2* gene with an Ala-encoding codon instead of the Met start codon) producing plasmid pCSMI44 that carries the fusion of the *gfp* gene to the 3′ end of *alr2310*. The resulting fusion was finally transferred as a KpnI-ended fragment to KpnI-digested pCSV3, which provides resistance to Sm and Sp (Valladares et al. 2011), producing pCSMI46. This plasmid, which bears the *alr2310*-*gfp* fusion gene, was transferred to *Anabaena* by triparental mating as described above, with selection for Sm^R^/Sp^R^. Insertion into *alr2310* and segregation of chromosomes carrying the fusion was confirmed by PCR analysis using template DNA from exconjugant clones and primers alr2310-13 and pRL500-1 for testing insertion of *gfp-mut2* and alr2310-13 and alr2310-16 for testing segregation of the mutated chromosomes. A homozygous clone bearing the *alr2310*-*gfp* construct was named strain CSMI21.

Complementation of the *alr2310* deletion mutant was performed with a replicating plasmid. Using DNA from *Anabaena* as template and primers alr2310-15/alr2310-16, which include SmaI sites close to their 5′ ends, ORF *alr2310* was amplified by PCR and ligated into pSpark (Canvax Biotech S.L., Córdoba, Spain) producing plasmid pCSMI53, whose insert was corroborated by sequencing. This insert was excised from pCSMI53 by digestion with SmaI and transferred to SmaI-digested pRL3845, replacing *all1711* by *alr2310*, producing pCSMI54. (pRL3845 is a Cm^R^ Em^R^-plasmid that contains a P_*glnA*_-*all1711* construct and can replicate in *Anabaena*; López-Igual et al. 2012.) As is the case for *all1711* in pRL3845, *alr2310* in pCSMI54 is expressed from the *Anabaena glnA* promoter. This plasmid was conjugated into strain CSMI11 (Δ*alr2310*) as described above, with selection for Em^R^, and tested for complementation of the mutant phenotype.

### Agmatinase assay in cell-free extracts

Filaments from 800 mL of cultures of *Anabaena* wild type and strain CSMI11 grown for 5 days in bubbled BG11 medium were harvested and washed with 10 mmol/L N-tris(hydroxymethyl)-methylglycine (Tricine)-NaOH buffer (pH 8.5) and resuspended with 100 mmol/L Tricine-NaOH buffer (pH 8.5) supplemented with 1 mmol/L dithiothreitol, 1 mmol/L MnCl_2_ and a protease inhibitor mixture tablet (cOmplete Tablets, Mini Ethylenediaminetetraacetic acid (EDTA)-free, Roche, Basel, Switzerland). Filament suspensions were passed twice through a French pressure cell at 20,000 psi. Cell debris was removed by centrifugation at 27,216*g*, 10 min at 4°C. The resulting supernatant constituted the cell-free extract from vegetative cells.

For determining agmatinase activity, cell-free extracts were supplemented with 5 mmol/L acetohydroxamic acid (a urease inhibitor) and incubated for 30 min at 30°C before adding 1 mmol/L agmatine sulfate (final concentration). The reaction was carried out at 30°C for 180 min. Reactions run without added agmatine were used as controls. Samples of 0.1 mL of the reaction mixture were taken at different times, supplemented with perchloric acid (final concentration, 5%), incubated at 0°C for 10 min and centrifuged at 16,100*g*, 5 min, at 4°C. The urea produced in the reaction was determined colorimetrically in 0.1 mL of the resulting supernatant by the method of Boyde and Rahmatullah (1980).

### Growth tests and nitrogenase activity

Protein concentration and chlorophyll *a* (Chl) content of the cultures or cell-free extracts were determined by a modified Lowry procedure (Markwell et al. 1978) and by the method of Mackinney (1941), respectively. The growth rate constant (*μ *= ln2/*t*_d_, where *t*_d_ is the doubling time) was calculated from the increase of protein content, determined in 0.2 mL samples, of shaken liquid cultures (Montesinos et al. 1995). Cultures were inoculated with an amount of cells containing about 5 *μ*g of protein mL^−1^ and grew logarithmically until reaching about 40 *μ*g of protein mL^−1^.

For growth tests in solid media, cultures grown in BG11_0_ NH_4_^+^ medium (supplemented with antibiotics when appropriate) were harvested and washed three times with 50 mL of BG11_0_ medium, and dilutions were prepared in BG11_0_ medium. Ten microliter samples of the resulting suspensions were spotted on agar plates with different nitrogen sources and incubated at 30°C in the light (25 *μ*E m^−2^ sec^−1^). When indicated, solid media were supplemented with filter-sterilized l-putrescine, agmatine sulfate or l-arginine purchased from Sigma-Aldrich (St. Louis, MO, USA).

Nitrogenase activity was determined by the acetylene reduction assay as described previously (Montesinos et al. 1995). Cells grown in 50 mL of BG11_0_ NH_4_^+^ medium were incubated 24 h without combined nitrogen (BG11_0_ medium) under growth conditions and used in the acetylene reduction assays performed under oxic or anoxic conditions. For the latter, the cell suspensions were placed in sealed flasks and supplemented with 10 *μ*mol/L 3-(3,4-dichlorophenyl)-1,1-imethylurea (DCMU), bubbled with argon for 3 min, and incubated for 90 min under assay conditions before starting the reaction by addition of acetylene.

### Cyanophycin measurements

To determine cyanophycin, filaments grown in BG11_0_ NH_4_^+^ medium were washed with BG11_0_ medium, inoculated at 0.5 *μ*g Chl mL^−1^ in 50-mL cultures with BG11_0_ NH_4_^+^ or BG11 media, and incubated for 8 days under growth conditions. Cyanophycin granule polypeptide (CGP) was isolated from filaments collected from these cultures. The filaments were harvested by centrifugation at 1,935*g* at room temperature, washed twice with, and resuspended in, sterile double destilled-purified H_2_O, and disrupted with a French pressure cell (two passages at 20,000 psi). After measuring the obtained volume of cell extract, Chl was determined in a 100-*μ*L sample. The remnant of each extract was centrifuged for 15 min, at 4°C and 27,216*g* and the pellets were washed twice with 11 mL of sterile milliQ-purified H_2_O and resuspended in 1 mL of 0.1 mol/L HCl for solubilization of CGP. After 2–4 h of incubation at room temperature and centrifugation under the same conditions, the resulting supernatants were removed and stored at 4°C. The pellets were resuspended in 1 mL of 0.1 mol/L HCl and incubated overnight at room temperature to complete solubilization of CGP, and the solution was subjected to centrifugation as above. The supernatants were combined with those obtained after the first centrifugation and stored at 4°C. These preparations were used for arginine guanidine group determination carried out by the Sakaguchi reaction as modified by Messineo (1966).

### Arginine catabolism

Cells grown in BG11_0_ NH_4_^+^ medium were harvested by centrifugation at 1,935*g* at room temperature, washed twice with 25 mmol/L Tricine–NaOH buffer (pH 8.1), and resuspended in the same buffer. The uptake assays were carried out at 30°C in the light (white light from fluorescent lamps, about 175 *μ*E m^−2^ sec^−1^) and were started by mixing a suspension of cells (2.1 mL) containing 5–10 *μ*g of Chl mL^−1^ with a solution (0.1 mL) of l-[U-^14^C]arginine (274 mCi mmol^−1^, from PerkinElmer, Waltham, MA, USA). The final concentration of arginine in the experiment was 1 *μ*mol/L. The rate of arginine uptake in the 10- and 30-min assays was estimated by taking a 0.5-mL sample of the cell suspension. The sample was filtered (0.45 *μ*m-pore-size Millipore HA filters were used) and the cells on the filters were washed with 5–10 mL of 5 mmol/L Tricine–NaOH buffer (pH 8.1). The filters carrying the cells were then immersed in 5 mL of scintillation cocktail, and their radioactivity was measured. Retention of radioactivity by boiled cells was used as a blank.

To determine metabolites produced from the labeled arginine at the end of the 10- and 30-min incubations, samples of 0.5 mL of the cell suspension were immediately (<15 sec) mixed, without filtering the cells, with 1.5 mL of water at 100°C and further incubated for 5 min in a bath of boiling water. The resulting suspensions were centrifuged, and samples (1–1.5 mL) from the supernatants were lyophilized and dissolved in 50 *μ*L of water. Samples of the resulting solutions were applied to 0.1-mm-thick cellulose thin-layer chromatography (TLC) plates (20 × 20 cm; Merck, Darmstadt, Germany). Two-dimensional separation of amino acids was effected by using the following solvents: the first dimension solvent consisted of *n*-butanol–acetone–ammonium hydroxide–water (20:20:10:4, vol/vol/vol/vol), and the second dimension solvent consisted of isopropanol-formic acid-water (20:1:5, vol/vol/vol). The TLC plates were analyzed by electronic autoradiography using a two-dimensional scanner for *β* particles (Cyclone Plus Phosphor Imager, PerkinElmer, Waltham, MA, USA), which allows a quantitative analysis of the radioactive spots. Identification of the metabolite originating a radioactive spot was made by co-chromatography by supplementing the samples with stable amino acids as markers and visualizing the amino acids after chromatography with a solution of ninhydrin in acetone in the presence of cadmium acetate (Atfield and Morris 1961).

### Determination of in vivo protein synthesis

To determine the radioactivity incorporated into macromolecules, a sample of the cell suspension in growth medium was incubated with 10 *μ*mol/L l-leucine supplemented with l-[U-^14^C]leucine (316 mCi mmol^−1^, from American Radiolabeled Chemicals, Inc., St. Louis, MO, USA). Samples of 0.5 mL were collected at different times and filtered to determine uptake into whole cells as above. Additionally, samples of 0.5 mL were added to ice-cold trichloroacetic acid (TCA; final concentration, 10%), incubated in ice-water for 60 min, and filtered as above. The filters were washed with 5 mL of ice-cold 10% TCA and immersed in a scintillation cocktail, and their radioactivity was measured.

### Western blot analysis

Filaments from 100 mL of cultures of *Anabaena* sp. strains PCC 7120 and CSMI21 grown in bubbled BG11 and BG11_0_ media were harvested, washed with buffer 1 (50 mmol/L Tricine-HCl buffer, pH 7.5, 10% glycerine, 100 mmol/L NaCl), and resuspended in 4 mL of the same buffer. To isolate heterocysts, 800 mL of cultures grown for 48 h in bubbled BG11_0_ medium were harvested and washed with buffer 2 (50 mmol/L imidazol and 0.5 mmol/L EDTA, pH 8.0), resuspended in 10 mL of the same buffer, incubated with 1 mg mL^−1^ of lysozyme for 15 min at room temperature and centrifuged at 1935*g* (4°C, 10 min). The pellets were resuspended in 5 mL of buffer 2 and passed three times through a French pressure cell at 3,000 psi. Samples were enriched in heterocysts after successive steps of low-speed centrifugation (200*g*, 10 min, 4°C) and washing with buffer 1, and the isolated heterocysts were resuspended in 4 mL of buffer 1. The filament and heterocyst suspensions were both supplemented with a protease inhibitor cocktail tablet (cOmplete Tablets, Mini EDTA-free, Roche) and passed two times through a French pressure cell at 20,000 psi. Cell debris was removed by centrifugation at 16,100*g* (4°C, 10 min). Proteins in the supernatants were resolved by SDS-PAGE and transferred to Hybond-P PVDF membranes (GE Healthcare, Buckinghamshire, England). Western analysis was performed incubating with an anti-GFP antibody (A6455 from Invitrogen, Carlsbad, CA, USA) diluted 1:2000 in Tween 20, Tris-buffered saline (TTBS) with 5% non-fat milk powder. Antigen–antibody complexes were visualized with a peroxidase-conjugated anti-rabbit-IgG antiserum (Sigma-Aldrich, St. Louis, MO, USA) and developed with the WesternBright™ Western blotting detection kit (Advansta, Menlo Park, CA, USA). A cell-free extract from *Anabaena* carrying pAM1954 (Yoon and Golden 1998) was used as a free GFP-containing control.

### Microscopy

Cells grown during 5–7 days in shaken liquid BG11_0_ NH_4_^+^ medium or heterocyst preparations were observed and photographed with a Zeiss (Oberkochen, Germany) Axioscop microscope equipped with a Zeiss ICc1 digital camera. GFP fluorescence was analyzed by confocal microscopy. Samples from cultures of *Anabaena* sp. strain CSMI21 or the wild-type PCC 7120 as a control grown in bubbled cultures with BG11 or BG11_0_ medium were visualized using a Leica HCX PLAN-APO 63X 1.4 NA oil immersion objective attached to a Leica TCS SP2 confocal laser-scanning microscope. GFP was excited using 488-nm irradiation from an argon ion laser. Fluorescence emission was monitored by collection across windows of 500–520 nm (GFP imaging) and 630–700 nm (cyanobacterial autofluorescence). Under the conditions used, optical section thickness was about 0.4 *μ*m. GFP fluorescence intensity was analyzed using ImageJ 1.43 m software (http://imagej.nih.gov/ij/).

## Results

### Construction and phenotype of an *alr2310* mutant

**Figure 2:**
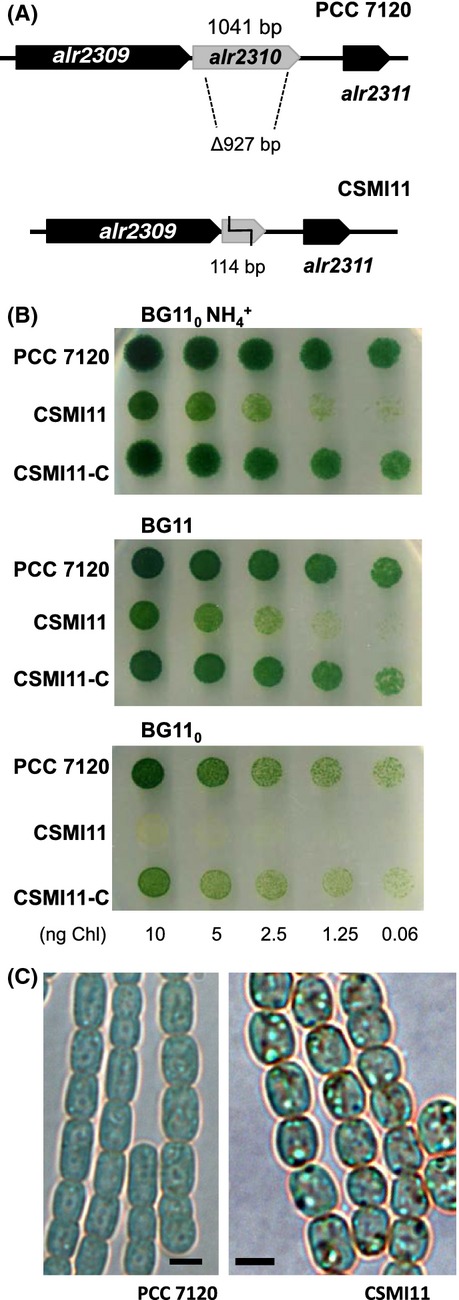
Characterization of an *Anabaena alr2310* mutant. (A) Schematic of the *alr2310* genomic region in *Anabaena* with indication of the DNA fragment removed to create strain CSMI11. (B) Growth tests in solid medium using ammonium (BG11_0_ NH_4_^+^), nitrate (BG11) or N_2_ (BG11_0_) as the nitrogen source. Each spot was inoculated with an amount of cells containing the indicated amount of Chl, and the plates were incubated under culture conditions for 7 days and photographed. Strain CSMI11-C is strain CSMI11 complemented with *alr2310*. (C) Filaments of *Anabaena* wild type (PCC 7120) and strain CSMI11 from cultures incubated for 5 days in BG11 medium and visualized by light microscopy. Scale bars, 2 *μ*m.

The *Anabaena* ORF *alr2310* is located between *alr2309* encoding a single-stranded nucleic acid binding protein and *alr2311* encoding an RNA-binding protein (Fig.2). Therefore, there is no obvious functional relation between the product of *alr2310* and those of its surrounding genes, and there is no evidence for co-transcription of these genes (Flaherty et al. 2011). Nonetheless, to investigate the role of the *alr2310* avoiding any possible polar effects of the mutation, a 927-bp in-frame internal fragment of the gene was removed without leaving any genetic marker in it (Fig.2A; see Fig. S1 and Materials and Methods for details). The Δ*alr2310* mutant, strain CSMI11, was homozygous for mutant chromosomes (Fig. S1). Tests in solid media showed that strain CSMI11 is impaired in growth in the presence of combined nitrogen (ammonium or nitrate) and is unable to grow under diazotrophic conditions (Fig.2B). In shaken liquid cultures, the growth rate constant of strain CSMI11 in ammonium- or nitrate-containing medium was, respectively, similar to or somewhat lower than that of the wild type, whereas the growth rate constant in combined *N*-free media was about 9% that of the wild type (Table1), confirming that strain CSMI11 is hampered in diazotrophic growth. Complementation of CSMI11 with a plasmid bearing wild-type *alr2310* allowed diazotrophic growth, corroborating that growth impairment resulted from the *alr2310* mutation (see strain CSMI11-C in Fig.2B). After incubation without a source of combined nitrogen, strain CSMI11 produced heterocysts that appeared immature (Fig. S2) and showed no nitrogenase activity, measured under both oxic and anoxic conditions (Table1). The production of aberrant heterocysts that lack nitrogenase activity can explain the impairment of strain CSMI11 in diazotrophic growth.

**Table 1:** Growth rate constants, nitrogenase activity, and cyanophycin granule polypeptide (CGP) levels in *Anabaena* sp. strains PCC 7120 (wild type) and CSMI11 (Δ*alr2310*).

Strain	Growth rate, μ (day^−1^)			Nitrogenase activity (*μ*mol [mg Chl]^−1^ h^−1^)		CGP (*μ*g arginine [mg Chl]^−1^)	
	NH_4_^+^	NO_3_^−^	N_2_	Oxic	Anoxic	NH_4_^+^	NO_3_^−^
PCC 7120	0.55 ± 0.08 (4)	0.79 ± 0.12 (8)	0.67 ± 0.22 (9)	7.13 ± 2.58 (3)	15.26 ± 3.05 (5)	115.12 ± 85.80 (3)	147.06 ± 6.61 (3)
CSMI11	0.56 ± 0.11 (4)	0.62 ± 0.12 (4)	0.06 ± 0.04 (4)	0.00 ± 0.00 (4)	0.003 ± 0.003 (3)	516.71 ± 123.89 (3)	1211.11 ± 429.58 (3)
*t* test	*P *=* *0.90	*P *=* *0.06	*P *=* *0.0003^*^	*P *=* *0.005^*^	*P *=* *0.0003^*^	*P *=* *0.02^*^	*P *=* *0.02^*^

The growth rate constant (*μ*) was determined in photoautotrophic shaken cultures with the indicated nitrogen source. To determine nitrogenase activity, filaments grown in BG11_0_ NH_4_^+^ medium and incubated in nitrogen-free BG11_0_ medium for 24 h were used in assays of reduction of acetylene to ethylene under oxic and anoxic conditions. Cyanophycin was determined by the Sakaguchi reaction for arginine on CGP granules isolated from ammonium- or nitrate-grown filaments. Figures are the mean and SD of data from the number of independent experiments indicated in parenthesis. The significance of the differences between the mutant and the wild-type figures was assessed by the Student's *t* test (*P* indicated in each case); asterisks denote likely significant differences.

Microscopic examination of strain CSMI11 showed the presence of abundant granulation in the cytoplasm of the cells growing in the presence of nitrate or ammonium (shown in Fig.2C for nitrate-grown filaments). To test whether that granulation corresponded to cyanophycin, CGP isolation was carried out and the isolated material was measured with the Sakaguchi reaction for arginine. CSMI11 cells grown for 8 days in the presence of nitrate or ammonium had, respectively, about 8.3- and 4.5-fold higher amounts of CGP than the control wild type cells (Table1).

### Arginine catabolism

Wild-type *Anabaena* and strain CSMI11 were used in uptake assays with [^14^C]arginine, and the fate of arginine was studied by TLC analysis of the radiolabeled compounds produced in filaments that had been incubated in BG11 or BG11_0_ medium. Consistent with results previously published for *Anabaena* (Montesinos et al. 1995), arginine was taken up at appreciable levels in filaments from either BG11 or BG11_0_ medium, and uptake was significant in both mutant CSMI11 and the wild type (Table2). Arginine was metabolized in both strains. After 30 min of incubation the amount of radioactivity that remained associated to arginine accounted for only 21.6 and 19.2% in BG11-grown cells of the wild type and the mutant, respectively, and for 50.3 and 17.4% in cells of the corresponding strain that had been incubated in BG11_0_ medium (Table2). Because metabolism was observed to proceed to a somewhat larger extent in mutant CSMI11 than in the wild type, catabolism of arginine is not inhibited by the inactivation of *alr2310*.

**Table 2:** l-[^14^C]arginine uptake and metabolic products in *Anabaena* sp. strains PCC 7120 (wild type) and CSMI11 (Δ*alr2310*).

	BG11				BG11_0_			
	PCC 7120		CSMI11		PCC 7120		CSMI11	
	10 min	30 min	10 min	30 min	10 min	30 min	10 min	30 min
Arginine taken up (nmol·[mg Chl]^−1^)	73.3	115.9	57.3	72.4	93.7	162.3	91.3	124.5
Labeled compounds (%)								
Origin	7.5	18.6	1.8	8.4	10.0	12.4	10.2	14.2
Arginine	54.8	21.6	52.6	19.2	66.7	50.3	51.1	17.4
Agmatine	0.4	0.4	2.9	10.4	0.2	0.3	7.1	15.4
Aspartate	1.5	1.5	2.1	1.8	0.5	0.8	1.1	1.2
Glutamate	7.0	33.0	24.6	52.0	1.6	10.7	8.6	37.1
Glutamine/Citrulline	6.4	3.2	3.7	1.2	2.5	2.0	1.8	1.1
Ornithine	2.2	1.0	1.6	1.2	2.5	1.6	2.4	1.5
Proline	15.4	17.4	8.6	2.7	9.5	15.8	9.3	6.4
Spot #6	4.6	2.5	2.1	1.5	6.6	5.1	8.4	3.3
Spot #7	–	0.8	–	1.7	–	1.3	–	2.4

Filaments of the indicated strains grown in BG11_0_ NH_4_^+^ medium and incubated for 24 h in BG11 or BG11_0_ medium were used at 5–10 *μ*g Chl mL^−1^ in assays of uptake of 1 *μ*mol/L l-[^14^C]arginine as described in Materials and Methods. After 10 and 30 min of incubation, uptake of arginine was determined and metabolites in the suspensions were analyzed by TLC. Cell-associated metabolites are presented as the percentage of the sum of radioactivity in ^14^C-labeled chromatographic spots after subtraction of extracellular arginine. Compounds that do not move with the used solvents accumulate in the origin of the chromatography.

**Figure 3:**
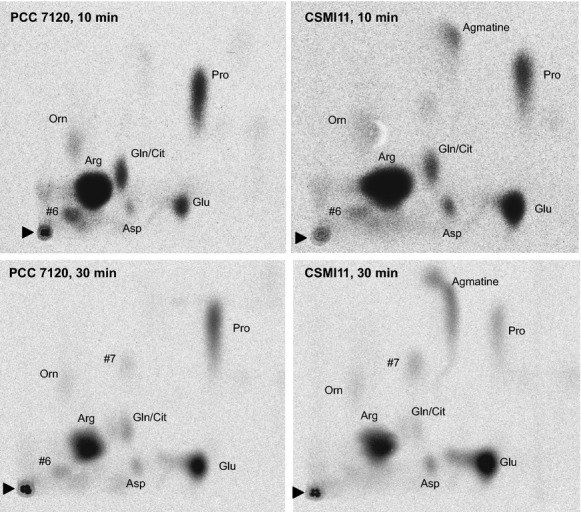
Production of ^14^C-labeled metabolites from l-[^14^C]arginine in filaments of *Anabaena* wild type (PCC 7120) and strain CSMI11 grown in BG11_0_ NH_4_^+^ medium and incubated in BG11 (nitrate-containing) medium for 24 h. Suspensions of filaments containing 5–10 *μ*g of Chl mL^−1^ were incubated for 10 and 30 min with 1 *μ*mol/L l-[^14^C-(U)]arginine. Metabolites in the cell suspensions were extracted and analyzed by TLC and autoradiography as described in Materials and Methods. The amino acids identified were as follows: arginine (Arg), citrulline (Cit), proline (Pro), glutamate (Glu), glutamine (Gln), ornithine (Orn), aspartate (Asp), and agmatine. Two unidentified spots, indicated as #6 and #7, were also detected. Note that the glutamine and citrulline spots overlap. The black triangles point to the origin of the chromatography.

In the suspensions of wild-type filaments from BG11 medium (Fig.3, Table2), after 10 min of incubation, radioactivity from [^14^C]arginine was mainly distributed among proline, glutamate, glutamine/citrulline (which overlap in the TLC system of solvents used in this study), ornithine, aspartate, and unidentified spot #6. After 30 min, an additional unidentified spot, #7, became visible, and a notable accumulation of radioactivity in glutamate and proline was observed. In strain CSMI11, the general pattern of labeled compounds was similar to that observed in the wild type but, remarkably, a spot that could be identified as agmatine was detected (Fig.3) that accounted for a significant 10.4% of the cell-associated radioactivity after 30 min of incubation. Additionally, substantially more radioactivity accumulated in glutamate, and less in proline, in strain CSMI11 than in the wild type.

In the suspensions of filaments that had been incubated in BG11_0_ medium, the patterns of [^14^C]arginine-derived products were similar to those found in the filaments from BG11 medium, although the accumulation of radioactivity in aspartate and, especially, in glutamate was lower in the filaments of both strains from BG11_0_ medium (Fig. S3; Table2). However, in the filaments from BG11_0_ medium, the accumulation of radioactivity in glutamate was also higher in the mutant than in the wild type (Table2). Additionally, a specific accumulation of agmatine, accounting for 15.4% of the cell-associated radioactivity, was also detected in filaments of the mutant from BG11_0_ medium (Fig. S3).

The results showing that agmatine accumulates specifically in mutant CSMI11 suggest that *alr2310* is an agmatinase. We therefore determined agmatinase in cell-free extracts from BG11-grown filaments as production of urea from agmatine. Whereas an activity of about 10 nmol urea produced (mg Chl)^−1^ h^−1^ was detected in extracts from the wild type, the activity was undetectable in extracts from strain CSMI11.

### Growth tests with arginine and arginine catabolism-related compounds

Alterations in growth of the Δ*alr2310* mutant, strain CSMI11 (lacking the activity of reaction 2 in Fig.1), could be explained by a lack of putrescine, which might be required as a polyamine or for production of other polyamines, or by the accumulation of agmatine, which might be detrimental to the cells. The effects of putrescine and agmatine on *Anabaena* were assessed by growth tests in solid medium, which were performed at a fixed pH of 7.5. Growth tests with different nitrogen sources and l-putrescine at 100 or 300 *μ*mol/L were performed, but no (or a very poor) recovery of the growth of strain CSMI11 in BG11_0_ medium was observed (Fig. S4). Although we cannot rule out that putrescine is not taken up by *Anabaena*, this possibility is unlikely because putrescine uptake has been reported in several cyanobacteria (Guarino and Cohen 1979; Raksajit et al. 2006) and the *Anabaena* genome bears genes encoding a putative polyamine ABC-type transporter (Kaneko et al. 2001).

**Figure 4:**
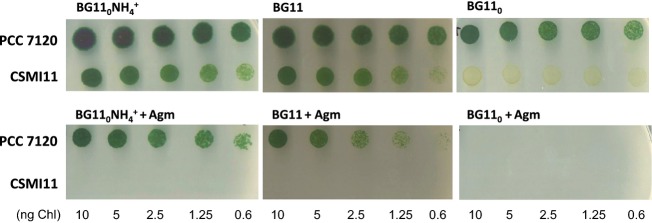
Growth test of *Anabaena* wild type (PCC 7120) and strain CSMI11 in BG11_0_ NH_4_^+^-, BG11-, and BG11_0_-solid media supplemented with 10 mM TES-NaOH buffer (pH 7.5) and, when indicated, 1 mmol/L agmatine sulfate (Agm). Each spot was inoculated with an amount of cells containing the indicated amount of Chl, and the plates were incubated for 8 days under culture conditions and photographed.

To test whether the impairment of strain CSMI11 in diazotrophic growth could result from an accumulation of agmatine, growth tests with different nitrogen sources and agmatine were performed. Whereas good growth was observed in the presence of 10 or 100 *μ*mol/L agmatine (not shown), 1 mmol/L agmatine was inhibitory for both the wild type and strain CSMI11, although in the presence of nitrate or ammonium the mutant was more sensitive than the wild type (Fig.4). The higher sensitivity of the mutant can be related to its inability to metabolize agmatine. In addition, these results show that the degree of agmatine toxicity depends on the nitrogen source.

**Figure 5:**
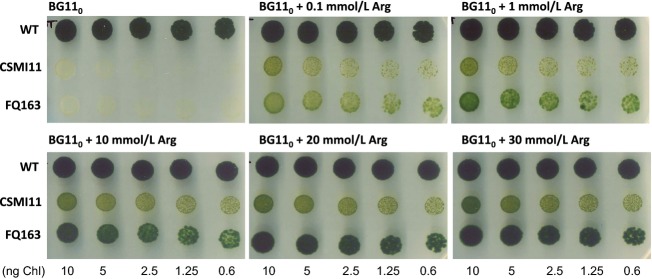
Growth tests of *Anabaena* sp. strains PCC 7120 (WT), CSMI11 (Δ*alr2310*) and FQ163 (*hepP*) in BG11_0_ solid medium supplemented with 10 mmol/L TES-NaOH buffer (pH 7.5) and, when indicated, arginine (Arg) at the specified concentration. Spots were inoculated with an amount of cells containing the indicated amount of Chl, and the plates were incubated for 14 days under culture conditions and photographed. Strain FQ163 was used as a Fox^−^ control.

To assess the possibility that agmatine is competing with arginine in cellular metabolism, we investigated whether the growth defect of strain CSMI11 in media lacking combined nitrogen could be rescued by arginine. Strain FQ163, a *hepP* gene mutant that is unable to grow fixing N_2_ under oxic conditions (Fox^−^ phenotype; López-Igual et al. 2012), was used as a control. Supplementation of the medium with 0.1–30 mmol/L arginine rescued the growth of both strains, CSMI11 and FQ163, but growth was stronger in the case of FQ163 than in the case of CSMI11 (Fig.5). Arginine can be utilized as a nitrogen source by *Anabaena* (Herrero and Flores 1990; Burnat et al. 2014), and it clearly serves as a nitrogen source for the Fox^−^ strain FQ163. In strain CSMI11, however, arginine compensates only to a limited extent the effect of inactivation of *alr2310*. It is possible that agmatine produced from arginine contributes to hamper growth.

### Protein synthesis in strain CSMI11

To determine if strain CSMI11 could be affected in protein synthesis, radioactivity incorporated from [^14^C] leucine into macromolecules was assessed. Leucine is an amino acid of low pool size that is primarily incorporated into protein, which makes it an appropriate amino acid to study protein synthesis in vivo (Padan et al. 1971). Cultures of wild-type *Anabaena* and strain CSMI11 were grown in BG11_0_ NH_4_^+^ medium, incubated for 24 h in BG11_0_ or BG11 medium and analyzed. As shown in Table3, leucine uptake was about 1.8-fold higher in strain CSMI11 than in the wild type when incubated in BG11 medium, whereas the rates of uptake were similar for both strains in BG11_0_ medium. Incorporation of radioactivity from [^14^C]leucine into TCA-precipitable material was 1.6-fold and 0.72-fold in strain CSMI11 as compared to the wild type when incubated in BG11 or BG11_0_ medium, respectively. Because the amount of [^14^C]leucine that is incorporated into protein can be expected to depend on the amount of [^14^C]leucine taken up, we compared the fraction of [^14^C]leucine taken up that was incorporated into TCA-precipitable material in the mutant and the wild type in each of the two growth conditions. Relative incorporation into TCA-precipitable material was about 13% lower in the mutant than in the wild type in BG11 medium, and about 25% lower in BG11_0_ medium. Therefore, the hampered diazotrophic growth of strain CSMI11 is hardly only a consequence of a low level of protein synthesis.

**Table 3:** [^14^C]Leucine uptake and incorporation into TCA-precipitable material in *Anabaena* sp. strains PCC 7120 (wild type) and CSMI11 (Δ*alr2310*).

Growth conditions	Strain	[^14^C]Leucine incorporation (nmol (mg Chl)^−1^ min^−1^)		TCA-precipitable material/uptake
		Uptake	TCA-precipitable material
BG11	PCC 7120	14.00 ± 8.59	11.91 ± 7.59	0.851
	CSMI11	25.03 ± 17.53	18.52 ± 13.43	0.740
BG11_0_	PCC 7120	41.50 ± 28.49	25.48 ± 14.07	0.614
	CSMI11	39.97 ± 32.40	18.44 ± 14.12	0.461

To determine leucine uptake (incorporation into whole cells) and the radioactivity incorporated into macromolecules, suspensions of filaments from cultures with BG11 or BG11_0_ medium were incubated in the same media with 10 *μ*mol/L l-[^14^C]leucine for 1 h, as described in Materials and Methods. At several time points, samples were filtered to determine leucine uptake rates or mixed with ice-cold TCA and then filtered to determine rates of incorporation into macromolecules. Figures are the mean ± SD of four independent experiments. For the fraction of radioactivity taken up that was precipitable with TCA, Student's *t* tests indicated that the differences between the mutant and the wild type were likely significant in both BG11 (*P *=* *0.006) and BG11_0_ (*P *=* *0.008) medium.

### Cell-specific expression of Alr2310

To investigate the expression and cell localization of Alr2310, an *alr2310-gfp* fusion gene was constructed and transferred to *Anabaena* (Fig. S5). *Anabaena* clones bearing this construct as the only *alr2310* gene were readily isolated. Strain CSMI21, which was selected for further analysis, exhibited growth properties similar to those of the wild type (Fig. S5), indicating that the Alr2310-GFP fusion protein retained Alr2310 function.

**Figure 6:**
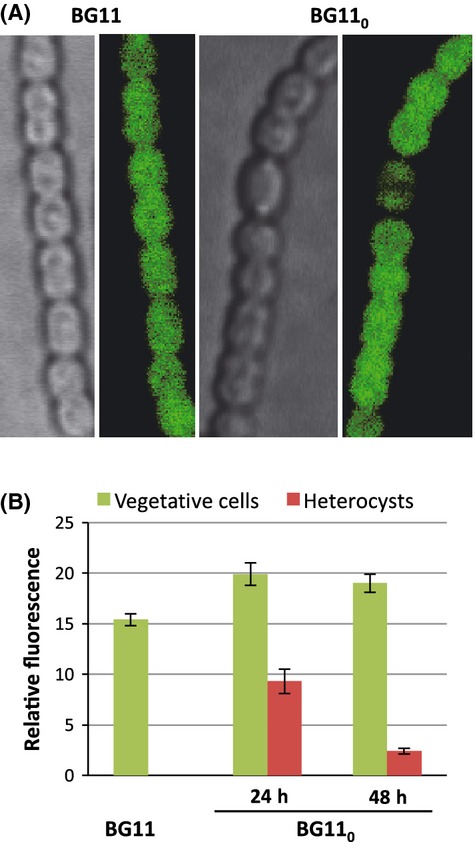
Cellular localization of Alr2310-GFP in the *Anabaena* filaments. (A) Filaments of *Anabaena* sp. strain CSMI21 grown in bubbled BG11 medium or incubated in bubbled BG11_0_ medium (without combined nitrogen) for 48 h were visualized by confocal microscopy as described in Materials and Methods. Bright field and GFP fluorescence images are shown Brightness and contrast were increased to improve visibility. (B) Quantification of GFP fluorescence in cells of strain CSMI21. Average background fluorescence from wild-type cells (which lack GFP) was subtracted. Figures are the mean and standard deviation of the mean of the fluorescence recorded in cells grown in bubbled BG11 medium (487 cells counted) or incubated in bubbled BG11_0_ medium for 24 h (309 vegetative cells and 58 heterocysts counted; Student's *t* test *P *=* *2.7 × 10^−18^) or 48 h (409 vegetative cells and 67 heterocysts counted; *P *=* *1.3 × 10^−56^).

When strain CSMI21 was grown with nitrate as the nitrogen source, GFP fluorescence was observed in all the cells of the filament, but when incubated in the absence of combined nitrogen, GFP fluorescence was observed in vegetative cells at higher levels than in heterocysts (Fig.6A). Quantification of the GFP fluorescence in vegetative cells and heterocysts indicated that in filaments incubated for 24 or 48 h without combined nitrogen, heterocysts had on average 46.7% and 12.6%, respectively, of the fluorescence detected in vegetative cells (Fig.6B).

**Figure 7:**
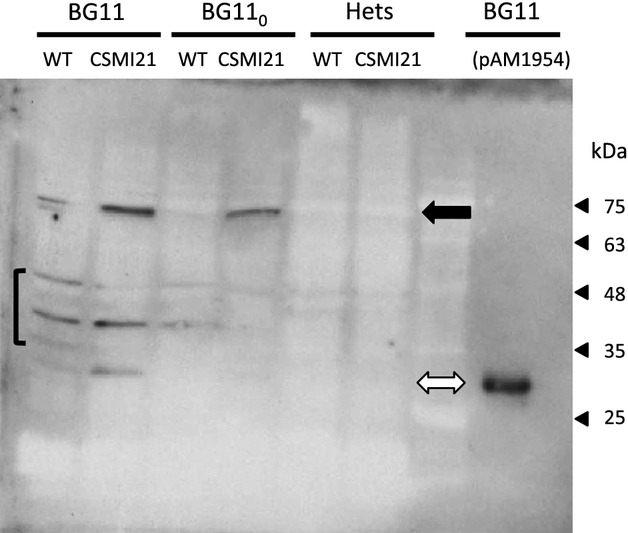
Western blot analysis of the GFP in strain CSMI21 and control strains. The *Anabaena* strains used were PCC 7120 (WT), mutant CSMI21 (*alr2310-gfp*) and a strain carrying pAM1954, which is a replicative plasmid bearing the *gfp* gene expressed from the *rbc* gene promoter. Cell-free extracts were prepared from whole filaments grown in bubbled cultures with BG11 medium or incubated in BG11_0_ medium for 48 h, or from heterocysts (Hets) isolated from the latter. *Anabaena* sp. [pAM1954] was grown in shaken cultures containing BG11 medium. Proteins were subjected to SDS-PAGE electrophoresis and transferred to membranes. Protein detection was performed with anti-GFP antibodies. Native GFP is 26.9 kDa and the Alr2310-GFP fusion, which includes Alr2310, a tetra-glycine linker peptide, and the GFP is 65.7 kDa. Protein loaded was 40 *μ*g per well (0.6 *μ*g in the case of *Anabaena* [pAM1954]). As observed with the wild-type extracts, the antibodies marked 3 or 4 unspecific bands (brackets). Strain CSMI21 produced mainly a band corresponding to the fusion protein (black arrow) and only a faint band corresponding to free GFP, which was identified with the extract of *Anabaena* [pAM1954] that only produced, as expected, free GFP (open arrow). Note that some material from lane BG11/CSMI21 may have contaminated lane BG11/WT.

Western blot analysis with anti-GFP antibodies identified a band corresponding to the Alr2310-GFP fusion protein in cell-free extracts from filaments of strain CSMI21 grown in bubbled BG11 medium or incubated in bubbled BG11_0_ medium for 48 h (Fig.7). Only a very low amount of free GFP released from the fusion protein was observed. Noteworthy, the fusion protein was not detected in cell-free extracts from heterocysts of strain CSMI21. The results of the western blot analysis are consistent with those of the GFP fluorescence analysis showing that the Alr2310-GFP fusion protein is present at significantly lower levels in heterocysts than in vegetative cells.

## Discussion

ORF *alr2310* from the genome of the heterocyst-forming cyanobacterium *Anabaena* putatively encodes an ureohydrolase family protein. Based on bioinformatic analysis, Alr2310 has been proposed to be the arginase of *Anabaena* (Schriek et al. 2007). However, as demonstrated by the accumulation of agmatine from [^14^C]arginine in the Δ*alr2310* mutant, strain CSMI11, and from lack of agmatinase activity in cell-free extracts from this mutant, *alr2310* encodes an agmatinase and therefore is the *speB* gene of *Anabaena*. Two possible agmatinases, Sll0228 and Sll1077, are encoded in the genome of *Synechocystis* sp. strain PCC 6803, and Alr2310 is most similar to Sll0228 (Schriek et al. 2007) whose inactivation also leads to lack of any agmatinase activity in *Synechocystis* cell-free extracts (Quintero et al. 2000). Accumulation of agmatine in strain CSMI11 in turn implies the operation of arginine decarboxylase, the product of ORF *all3401*, in *Anabaena* (Fig.1). We have not consistently detected in the wild type a TLC spot that could be identified as putrescine. We do not know, however, whether in our experiments the radioactivity from putrescine could pass to other metabolites or whether our methodology is inadequate to extract this polyamine.

Cells of the CSMI11 mutant grown with combined nitrogen (nitrate or ammonium) show an extensive granulation in the cytoplasm (Fig.1C) that correlates with the accumulation of CGP (Table1). It has been reported that the end products of cyanophycin mobilization, arginine and aspartate, inhibit cyanophycinase (Gupta and Carr 1981a). Agmatine is known to inhibit moderately cyanophycin synthetase (Aboulmagd et al. 2001), but we do not know whether it could also inhibit cyanophycinase as to account for the observed accumulation of CGP in the CSMI11 mutant. Alternatively, if, as is the case for amino acids such as lysine, glutamate, citrulline and ornithine (Merrit et al. 1994; Ziegler et al. 1998; Berg et al. 2000; Aboulmagd et al. 2001), agmatine were incorporated into cyanophycin at significant levels, accumulation of CGP could result from an inefficient degradation of agmatine-containing cyanophycin.

Strain CSMI11 is impaired in diazotrophic growth and produces only immature heterocysts, and CSMI11 filaments incubated for 24 h without combined nitrogen do not show measurable nitrogenase activity (Table1). Inactivation of *alr2310* likely results in accumulation of agmatine (as observed with [^14^C] arginine) and a lack of putrescine, but growth inhibition appears to be related specifically to accumulation of agmatine. Inhibition of the growth of some bacteria by agmatine has previously been noted, and competitive inhibition of amino acid transport or interference with translation were considered as possible mechanisms of inhibition (Griswold et al. 2006). Agmatine may compete with arginine for charging tRNAs resulting in a general inhibition of protein synthesis. We have found that incorporation of [^14^C]leucine into macromolecules is somewhat inhibited in strain CSMI11 as compared to the wild type, especially in cells that had been incubated in the absence of combined nitrogen, but the observed effect seems insufficient to prevent growth. Because diazotrophic growth of *Anabaena* requires heterocyst differentiation and, as mentioned earlier, strain CSMI11 produces only immature heterocysts, interference of agmatine with heterocyst differentiation may contribute to prevent growth of this mutant in the absence of combined nitrogen.

Because the pattern of [^14^C]arginine metabolic products in *Anabaena* is very similar to that reported for *Synechocystis* sp. strain PCC 6803 (Quintero et al. 2000), it is possible that the arginine catabolism pathway proposed for this cyanobacterium, which combines the arginase catabolic route and a urea cycle generating proline, glutamate and glutamine as final products (Quintero et al. 2000), is operative also in *Anabaena*. An interesting aspect of this pathway is that it incorporates aspartate at the level of the urea cycle (Fig.1), thus providing a rationale for the combined utilization of the amino acids released in cyanophycin mobilization, arginine and aspartate. Appreciable levels of arginase activity have been detected in cell-free extracts of *Anabaena* (Gupta and Carr 1981b), but the gene encoding an enzyme with arginase activity remains to be identified in this cyanobacterium. The possibility that Alr2310 has also arginase activity is not supported by the results of inactivation of *alr2310*, which does not prevent production of proline and glutamate from [^14^C]arginine. Instead, production of glutamate from [^14^C]arginine is significantly increased in mutant CSMI11 as compared to the wild type (Table2). This result suggests that if the arginine decarboxylase pathway is not operative, arginine degradation through the arginase-like pathway is increased. A small amount of [^14^C]aspartate produced from [^14^C]arginine could also be observed in our experiments, but we do not know the possible pathway involved in this conversion.

As described in the Introduction, we have recently found that in the diazotrophic filament of *Anabaena,* the second step of cyanophycin degradation, hydrolysis of *β*-aspartyl-arginine, takes place mainly in vegetative cells (Burnat et al. 2014). This dipeptide appears to be an important vehicle for the transfer of nitrogen from heterocysts to vegetative cells, consistent with the possible existence of a gradient of arginine or an arginine-containing compound in the diazotrophic filament (Ke and Haselkorn 2013). These results imply an active catabolism of arginine (and aspartate) in the vegetative cells of the diazotrophic filament. Consistently, arginase and a low level of arginine deiminase activity detected in *Anabaena* are present at significantly higher levels in vegetative cells than in heterocysts (Gupta and Carr 1981b). Our results showing the presence of the Alr2310-GFP fusion protein at significantly higher levels in vegetative cells than in heterocysts indicate that also agmatinase, and hence the arginine decarboxylase pathway, is mainly operative in the vegetative cells of the diazotrophic filament. These observations highlight the importance of arginine catabolism to make available in the vegetative cells the nitrogen transiently stored as cyanophycin and transferred from the heterocysts.

## Supporting Information

Figure S1 Construction and verification of mutant CSMI11.

Figure S2 Microscopic observation of filaments of mutant strain CSMI11.

Figure S3 Catabolism of L-[^14^C]arginine in filaments of *Anabaena* wild type and strain CSMI11 incubated in medium lacking combined nitrogen.

Figure S4 Growth tests of *Anabaena* wild type and strain CSMI11 in solid media supplemented with putrescine.

Figure S5 Construction, verification and growth test of strain CSMI21.

Table S1 Cyanobacterial strains and plasmids used in this work.

Table S2 Oligodeoxynucleotide primers used in this work.
