# Supplementary material for: Inactivation of agmatinase expressed in vegetative cells alters arginine catabolism and prevents diazotrophic growth in the heterocyst-forming cyanobacterium Anabaena
Source: Microbiologyopen. 2014 Sep 10;3(5):777–92. doi: 10.1002/mbo3.207 (PMC4234267; doi:10.1002/mbo3.207)
Supplement: Table S1 — Cyanobacterial strains and plasmids used in this work. [file mbo30003-0777-sd6.docx]

**Table S1.** Cyanobacterial strains and plasmids used in this work.

| Strain or plasmid | Relevant characteristic(s) | Source or reference |
| --- | --- | --- |
| ***Anabaena* strains** |  |  |
| PCC 7120 | Wild type | 1 |
| FQ163 | *hepP(all1711)*::Tn5-1063; Nm^R^ | 2 |
| CSMI11 | Δ*alr2310* | This study |
| CSMI11-C | Δ*alr2310* bearing wild type *alr2310* in replicative plasmid pCSMI54; Em^R^Cm^R^ | This study |
| CSMI21 | *alr2310*-C-*gfp*; Sm^R^Sp^R^ | This study |
|  |  |  |
| **Plasmids** |  |  |
| pMBL-T | Cloning vector; Ap^R^ | Dominion MBL |
| pSPARK | Cloning vector; Ap^R^ | Canvax, Biotech SL |
| pRL278 | *sacB*-containing negative selection vector with: Km^R^ Suc^S^ | 3 |
| pRL443 | Conjugative plasmid; Ap^R^ | 4 |
| pRL623 | Helper plasmid; carries *mob* and DNA methylases; Cm^R^ | 5 |
| pCSRO | *sacB*-containing negative selection vector with: Sm^R^Sp^R^ Suc^S^ | 6 |
| pCSV3 | Positive selection vector; Sm^R^Sp^R^ | 7 |
| pAM1954 | Plasmid carrying the fusion P*_rbcL_::gfp* | 8 |
| pRL3845 | Replicative vector used for complementation; Em^R^Cm^R^ | 2 |
| pCSBN1 | Positive selection vector derived from pRL278 and pCSV3; Km^R^ Suc^S^ | This study |
| pCSBN4 | *alr2310* lacking a 910-bp internal fragment cloned in pMBL-T; Ap^R^ | This study |
| pCSBN5 | SacI fragment from pCSBN4 cloned into SacI-digested pCSBN1; Km^R^ Suc^S^ | This study |
| pCSMI42 | 681-bp fragment from the 3’ part of *alr2310* cloned in pMBL-T; Ap^R^ | This study |
| pCSMI44 | SacI/NheI fragment from pCSMI42 cloned into SacI/NheI-digested pCSAL33; Ap^R^ | This study |
| pCSMI46 | KpnI fragment from pCSMI44 cloned into KpnI-digested pCSV3; Sm^R^Sp^R^ | This study |
| pCSMI53 | *alr2310* cloned in pSPARK; Ap^R^ | This study |
| pCSMI54 | SmaI fragment from pCSMI53 cloned into SmaI-digested pRL3845; Em^R^Cm^R^ | This study |
| pCSAL33 | Plasmid carrying *gfp;* Ap^R^ | A. López-Lozano and A. Herrero |

^R^ denotes resistance to the indicated antibiotic: Ap, ampicillin; Cm, chloramphenicol; Em, erythromycin; Km, kanamycin; Nm, neomycin; Sm, streptomycin; and Sp, spectinomycin. Suc^S^, sensitivity to 5% sucrose.

1. Rippka, R., Deruelles, J., Waterbury, J.B., Herdman, M., Stanier, R.Y. (1979) Generic assignments, strain histories and properties of pure cultures of cianobacteria. *J Gen Microbiol* **111**: 1-61
2. López-Igual, R., Lechno-Youssef, S., Fan, Q., Herrero, A., Flores, E., Wolk, C.P. (2012) A major facilitator superfamily protein, HepP, is involved in formation of heterocyst envelope polysaccharide in the cyanobacterium *Anabaena* sp. strain PCC7120. *J Bacteriol* **194**: 4677-4687
3. Black, T.A., Cai, Y., Wolk, C.P. (1933) Spatial expression and autoregulation of *hetR*, a gene involved in the control of heterocysts development in *Anabaena*. *Mol Microbiol* **9**:77-84
4. Elhai. J., Vepritskiy, A., Muro-Pastor, A.M., Flores, E., Wolk, C.P. (1997) Reduction of conjugal transfer efficiency by three restriction activities of *Anabaena* sp. strain PCC 7120. *J Bacteriol* **179**: 1998-2005.
5. Elhai, J., Wolk, C.P. (1988) Conjugal transfer of DNA to cyanobacteria. *Methods Enzymol* **167:** 747-754.
6. Merino-Puerto, V., Herrero, A., Flores, E. (2013) Cluster of genes that encode positive and negative elements influencing filament length in a heterocyst-forming cyanobacterium. *J Bacteriol* **195**: 3957-3966
7. Valladares, A., Rodríguez, V., Camargo, S., Martínez-Noël, G.M.A., Herrero, A., Luque, I. (2011) Specific role of the cyanobacterial PipX factor in the heterocysts of *Anabaena* sp. strain PCC 7120. *J Bacteriol* **193**: 1172-1182
8. Yoon, H.S., Golden, J.W. (1998) Heterocyst pattern formation controlled by a diffusible peptide. *Science* **282**:953-938
