# Supplementary material for: Inactivation of agmatinase expressed in vegetative cells alters arginine catabolism and prevents diazotrophic growth in the heterocyst-forming cyanobacterium Anabaena
Source: Microbiologyopen. 2014 Sep 10;3(5):777–92. doi: 10.1002/mbo3.207 (PMC4234267; doi:10.1002/mbo3.207)
Supplement: Table S2 — Oligodeoxynucleotide primers used in this work. [file mbo30003-0777-sd7.docx]

**Table S2.** Oligodeoxynucleotide primers used in this work.

| **Primer name** | **Sequence (5’ to 3’)** |
| --- | --- |
| alr2310-3 | GAGCTCCCAGGCCCCAACGGTGAAG |
| alr2310-4 | GAGCTCCAAAATCATGGCTGTGTAGAGA |
| alr2310-5 | CCTAGCGATATCGAATGGGATGGTAATGTA |
| alr2310-6 | CCATTCGATATCGCTAGGGTTATAGTCTTG |
| alr2310-7 | ATGCGTCCATGGGATTCCTCAA |
| alr2310-8 | GCACAACCCGTTTTACAGAACCA |
| alr2310-11 | CAAGCTTCCCAACAGGTAAATCAATGGCTG |
| alr2310-12 | GCTAGC*ACCTCCACCGCC*TTGTTTGTGAGATAAATC |
| alr2310-13 | CACTGCACGTTTACCCTTATGGTGTGAGTAGG |
| alr2310-15 | TGCCCGGGTTTGTCGAGTAAATCCA |
| alr2310-16 | TTCCCGGGTGTCTAGGCAATGACTGA |
| SacB-1 | CTTGAGGTACAGCGAAGTG |
| SacB-2 | TCTGCAAAAGGCCTGGAGG |
| Universal | GTAAAACGACGGCCAGT |
| M13REV | CAGGAAACAGCTATGAC |
| pRL500-1 | ATAGGCGTATCACGAGGC |

Introduced restriction enzyme cutting sites are underlined. The nucleotide sequence that, inverted, encodes the four-Glycine linker is in italics.
